# Initial allergenicity assessment of *Ulva* sp. seaweed flour

**DOI:** 10.1038/s41538-025-00638-x

**Published:** 2025-12-03

**Authors:** Odeya Kedar, Alexander Golberg, Uri Obolski, Efraim Steinbruch, Alexander Chemodanov, Álvaro Israel, Yoav D. Livney, Idit Lachover-Roth, Yossi Rosman, Ronit Confino-Cohen

**Affiliations:** 1 School of Mechanical Engineering, Faculty of Engineering, Tel Aviv University, Tel Aviv, Israel; 2 Department of Epidemiology and Preventive Medicine, School of Public Health, Gray Faculty of Medical & Health Sciences, Tel Aviv University, Tel Aviv, Israel; 3 Israel Oceanographic and Limnological Research Ltd. (PBC), Tel Shikmona, Haifa, Israel; 4 Faculty of Biotechnology and Food Engineering, The Technion – Israel Institute of Technology, Haifa, Israel; 5 Allergy and Clinical Immunology Unit, Meir Medical Center, Kfar Saba, Israel; 6 School of Medicine, Gray Faculty of Medical & Health Sciences., Tel Aviv University, Tel Aviv, Israel

**Keywords:** Plant sciences, Environmental sciences, Health care, Risk factors, Science, technology and society

## Abstract

*Ulva* sp. is a green marine macroalga and a promising yet underexplored sustainable food source, with limited research on its allergenicity. This study evaluated the allergenic potential of *Ulva* sp. demineralized biomass flour (Ulva-DBF) using a weight-of-evidence approach inspired by regulatory assessments for novel foods, combining literature review, proteomic analysis, and a pilot clinical trial. Literature revealed no documented *Ulva* allergenicity. Proteomic analysis identified a few putative allergens, with estimated allergenic potential below 0.02%. In a pilot clinical trial (registered at ClinicalTrials.gov, Identifier: NCT06452381; First Posted Date: June 11, 2024), 31 participants consumed Ulva-DBF. No allergic reactions occurred, although 16.6% reported gastrointestinal discomfort. These findings represent a preliminary evaluation phase before widespread exposure. Nevertheless, large-scale consumption remains the most effective way to assess public health risks associated with any food. As we seek innovative and sustainable food solutions, post-marketing monitoring is vital to ensure their safety.

## Introduction

The increasing demand for new food sources to address global population growth, sustainability, environmental challenges, and animal welfare is driving the search for sustainable, plant-based alternatives^1^. Among these, the green marine macroalga *Ulva* sp. presents a promising, yet underutilized option. With its widespread availability, high protein content^2^, and associated nutritional benefits^3^, *Ulva* has significant potential in the food production chain^4,5^. Although it is not classified as a novel food due to its traditional consumption in Asia^6^ and Europe^7^, which permits its market use, it remains scarce in many global markets, hence potentially new to numerous consumers. To broaden its application in food production, it is important to evaluate its potential allergenicity alongside other aspects. This study focuses on demineralized biomass flour derived from *Ulva sp*. (Ulva-DBF), investigating its allergenic potential.

In the absence of a definitive allergenicity test, we adopted a weight-of-evidence approach, inspired by regulatory frameworks, such as the U.S. Food and Drug Administration (FDA) and the European Food Safety Authority (EFSA)^8^. This included a literature review, an in silico homology comparison of *Ulva*’s protein compositions with known allergens^8^, and a pilot clinical trial involving an oral food challenge (OFC). While clinical trials are rarely used for regulatory purposes, they offer irreplaceable insights by capturing the complexity of immune responses triggered by whole food consumption, which cannot be replicated by isolated laboratory tests. This study aims to advance understanding of *Ulva* as a potential common food source, providing a comprehensive risk assessment of its allergenicity and raising awareness about the allergenic risks associated with novel or less familiar foods.

## Results

The genus *Ulva* comprises 128 species, several of which are commercially produced using diverse methods^9–11^. Several species have been consumed for centuries, particularly in East Asia and northern coastal regions^6^. However, recent molecular techniques have highlighted gaps in the understanding of *Ulva*’s genetic diversity, presenting taxonomy challenges^12^. *Ulva* sp. is also recognized for its environmental benefits, such as bioremediation^13^ and carbon dioxide sequestration^7^. As a food source^14^, *Ulva* sp. demonstrates high nutritional quality, characterized by a high protein concentration with good quantities of essential amino acids^15,16^, rich mineral content, and elevated levels of functional polysaccharides and essential fatty acids^17^. In our laboratory, Ulva-DBF flour was found to contain 35% protein, 1% lipids, 17% ash, and 47% carbohydrates (calculated by difference; see Supplementary Information for supporting document). However, allergenicity-related concerns exist about *Ulva* grown in seawater being exposed to fouling organisms, such as crustacean shellfish. Therefore, packaged macroalgae products grown in seawater must be labeled with a crustacean shellfish allergen warning (major allergens)^18^. Additionally, toxicity-related concerns arise from *Ulva* being exposed to pollutants from seawater, such as heavy metals and pesticides^19^. Nonetheless, *Ulva* consumption has been shown to be generally safe^6,20^, and potential risks can be mitigated through enclosed cultivation^21^. Furthermore, the fermentation of *Ulva* by colonic bacteria^22,23^, and associated sulfate reduction, requires further investigation^23,24^.

The in silico study identified 28 potential allergen peptides in *Ulva lactuca* using AllerTop version 2.0 (http://www.ddg-pharmfac.net/AllerTOP)^25^. AllerTop employs methods divergent from regulatory standards and can yield contradictory results; therefore, while we report the findings, we do not endorse them. Additionally, another in silico study from our lab identified various homologous food allergens, including Troponin C and manganese superoxide dismutase (MnSOD), all showing homology scores below 41%^26^.

The search terminology used for allergy research, such as in PubMed Central®, yielded limited results: “*Ulva* AND allergenicity” (3 results), “*Ulva* AND allergy” (8 results), and “macroalgae AND allergenicity” (22 results). Recent publications have identified only a few cases of allergic reactions, all associated with red macroalgae, and no clinical reports of allergy to green seaweeds and specifically to *Ulva* sp. or macroalgae-specific proteins^14,27^. A single case of allergenicity to red seaweeds (Nori) was confirmed based on clinical history and a 13-mm wheal in a skin prick test (SPT), while tests for *Ulva* species and crustaceans were negative^28^. Furthermore, two documented cases suggest rare IgE-mediated hypersensitivity reactions to polysaccharides, particularly carrageenan, a food additive derived from red macroalgae^29,30^. Another study observed IgE antibodies to various gums, including carrageenan, in healthy individuals without allergic symptoms^31^. Finally, red seaweed extract and agar-agar showed positive results in the SPT of an individual allergic to antacid tablets^32^. Notably, studies on Ulva-derived polysaccharides have shown anti-allergic effects in a murine food allergy model^33^.

**Fig. 1:**
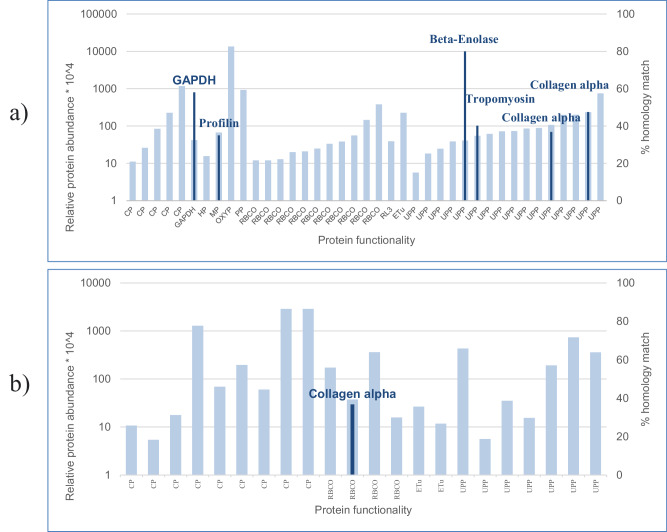
Allergenic protein differences in Ulva before (Ulva-UW) and after processing (Ulva-DBF). **a** Proteins detected uniquely in Ulva-UW. 39 proteins were detected uniquely in Ulva-UW, with non-zero Intensity-Based Absolute Quantitation (iBAQ) values in Ulva-UW but absent (zero iBAQ) in Ulva-DBF. Several of these proteins showed homology to known food allergens, indicating that processing may reduce the abundance of potentially allergenic proteins. **b** Proteins detected uniquely in Ulva-DBF. 22 proteins were detected uniquely in Ulva-DBF, with non-zero iBAQ values in Ulva-DBF but absent in Ulva-UW. Among these, one protein was homologous to the Collagen alpha allergen. Light blue bars denote proteins detected in Ulva-UW and Ulva-DBF, respectively, and dark blue bars represent proteins with allergen homology. Allergen names are shown within the bars for clarity.

Proteomic analysis of Ulva-DBF and Ulva-UW identified 302 proteins for homology comparison. This analysis generated Fig. 1, Table 1, and Supplementary Data 1. Figure 1 illustrates changes in protein composition resulting from the washing step and gamma irradiation. Proteomic analysis, compared against an allergen database, confirmed that a protein homologous to profilin was completely removed from Ulva-DBF during these processes, while other allergens showed multiple homologies with proteins retained in Ulva-DBF (Supplementary Data 1). Supplementary Data 1 lists all Ulva-DBF proteins with homology scores to food allergens exceeding 35%, detailing 22 homologous food allergens and their corresponding Ulva-DBF proteins, primarily sourced from Allergen.org. Notably, four proteins exhibited homology above 70%, as summarized in Table 1: glyceraldehyde-3-phosphate dehydrogenase (GAPDH), cyclophilin, beta-enolase, and MnSOD.

**Table 1:** Ulva-DBF food allergen assessment summary with AOL Best 80mer sliding window homology match above 70%

Allergen counter	Allergen source	Ulva-DBF protein Accession Number (biochemical name)	Relative protein abundance (%)	Best 80mer sliding window homology match (%)	Full alignment homology match (%)	Official Allergen.org name (created)	AOL allergenicity
1	Striped catfish	QZI95758.1 (GAPDH, partial)	0.007	82.50	68.40	Pan h 13 (2019)	IgE, no biological test
2	Peanuts	ABR23154.1 (cyclophilin)	0.267	80.00	72.80	Ara h 18 (2015)	IgE, no biological test
3	Salmon	CAD7700860.1 (unnamed protein product)	0.000	80.00	66.30	Sal s 2 (2011)	IgE + basophil and/or SPT
3	Yellowfin tuna	CAD7700860.1 (unnamed protein product)	0.000	80.00	64.40	Thu a 2 (2012)	IgE + basophil and/or SPT
3	Striped catfish	CAD7700860.1 (unnamed protein product)	0.000	77.52	67.00	Pan h 2 (2019)	IgE, no biological test
3	Chicken	CAD7700860.1 (unnamed protein product)	0.000	77.52	63.60	Gal d 9 (2016)	IgE + basophil and/or SPT
4	Pistachio	ABR23158.1 (MnSOD)	0.082	70.00	55.30	Pis v 4 (2007)	IgE, no biological test

*AOL* AllergenOnline, *GAPDH* glyceraldehyde-3-phosphate dehydrogenase, iBAQ intensity-based absolute quantitation, MnSOD manganese superoxide dismutase, Allergen.org nomenclature database.

Studies have identified **GAPDH** as a potential allergen in shrimp^34,35^ and the Bombay locust, with the latter suggested to have increased GAPDH allergenic properties after food processing^36^. Additionally, GAPDH was identified as an in vitro minor allergen in catfish^37^ and *M. rosenbergii*^35^. However, Allergen.org mentions only three GAPDH allergens, with striped catfish as the sole source of food allergens. Clinical data indicate that 6% of fish-allergic patients had relevant IgE to GAPDH (Supplementary Data 1).

**Cyclophilins** widely found in microorganisms and in organisms, including mammals, and are known for their ubiquity and conservation. While recognized as pan-allergens^38^, plant Cyclophilins do not cross-react with non-plant counterparts^39^. Therefore, discussing allergens from sources like house dust mites, mice, or shrimp may not directly apply to *Ulva* sp.^34,40^. Tomato cyclophilin, mentioned alongside peanut in Allergen.org, may be more relevant as a food allergen. Clinical data for this allergen are limited, and it is potentially cross-reactive with pollen and respiratory allergens^41^. Among peanut-allergic patients, 4% showed relevant IgE responses to Cyclophilins (Supplementary Data 1). Homologous matches for protein ABR23154.1 were found in the AllergenOnline (AOL) database, showing 81.3% homology to carrot cyclophilin and 80% to peanut cyclophilin. Given the lack of recorded allergenicity for carrot cyclophilin, we prioritized the peanut match in Table 1 and Supplementary Data 1.

CAD7700860.1 exhibits sequence homology to 4 **beta-enolases**: 3 from fish and 1 from chicken. However, its post-wash amount in Ulva-DBF is undetectable, suggesting that its concentration falls below the limit of quantitation^42^. This protein is labeled an “unnamed protein product,” possibly indicating beta-enolases.

**MnSOD** is identified as a pan-allergen, binding IgE in fungi, humans, yeast, *Drosophila*, and *E. coli*^43^. Its significance as an in vitro major or minor allergen in pistachios varies, probably due to small serum sample sizes^44^. Pistachio is the sole source of MnSOD as a food allergen (Allergen.org).

How many participants are needed to find clinical evidence of allergic reactions to Ulva-DBF, according to the proteomic analysis? We identified three homologous allergens with sequence identities exceeding 70%. Prevalence calculations (Table 2) based on local allergy data^45^ and sera IgE levels (Supplementary Data 1), suggest a potential allergenic prevalence of 0.01884% (0.0054% + 0.0056% + 0.00784%, as shown in Table 2). Notably, food allergy prevalence among young adults in Israel is lower than in other Western countries^45^. Variability in regional sera IgE prevalence among allergic groups could lead to different results across populations. However, for evaluation purposes, these values are assumed to represent general orders of magnitude.

**Table 2:** Prevalence of Ulva-DBF allergens with > 70% homology to known allergens in the local population

Allergen homologous source	Allergy prevalence	Relative protein abundance	Sera IgE of relevant allergic participants	Specific allergen prevalence in the general population
Pan h 13 GAPDH from Striped catfish	Fish allergies: 0.09%^45^	0.007%	6% had IgE binding to raw catfish GAPDH^37^	0.09% × 6% = 0.0054%
Ara h 18 Cyclophilin from Peanuts	Peanut allergies: 0.14%^45^	0.267%	4% had IgE binding to Ara h 18^69^	0.14% × 4% = 0.0056%
Pis v 4 MnSOD from Pistachio	Pistachio allergy affects 7% of tree nut allergy cases^58^ (0.28%)^45^, 0.28% × 7% = 0.0196%	0.082%	40% had IgE binding to Pis v 4^70^	0.0196% × 40% = 0.00784%

*GAPDH* glyceraldehyde-3-phosphate dehydrogenase, *MnSOD* manganese superoxide dismutase.

All 31 participants were initially enrolled in the Ulva-DBF clinical trial. No participants were excluded due to SPT sensitivity to Ulva-DBF or other tested allergens. However, 10 participants did not complete the study: 5 due to abdominal symptoms, 2 because of dissatisfaction with the taste and texture of Ulva-DBF, 2 for personal reasons, and 1 due to worsening of a preexisting thyroid condition. After one month of avoidance, 21 participants underwent SPT and an open oral Ulva-DBF challenge. All participants who completed follow-up (*n* = 21) were included in the analysis. No sensitization or allergic reactions were observed during testing.

## Discussion

This study evaluated the allergenicity of *Ulva* sp., processed into flour (‘Ulva-DBF’), using a weight-of-evidence approach inspired by regulatory standards for novel food. The literature review indicated that allergic reactions to macroalgae are mostly associated with red species. Since red and green macroalgae belong to distinct polyphyletic groups (with no recent common ancestor), cross-reactivity is unlikely^28^. Among reported cases, two rare allergic reactions to carrageenan^29,30^ and one suspected case involving agar-agar^32^ were documented. Ulva-DBF contains the sulfated polysaccharide ulvan, which, similarly to carrageenan and agar-agar, can be utilized for gelling and stabilizing^46^. Notably, no allergic reactions to ulvan have been reported. However, the discomfort observed during the clinical trial may be attributable to the high fiber content of Ulva-DBF, and/or the presence of this sulfated polysaccharide.

Our proteomic process focused on the final Ulva-DBF product. However, regulatory assessments often target specific proteins due to factors such as genetic manipulation,^8^ protein abundance^47^, or the presence of major allergens^48^, none of which were relevant here. Academic research frequently relies on DNA^49^ and RNA^34^ data; however, Ulva’s full genome remains unsequenced. Moreover, *Ulva* is cultivated in open-air environments, exposed to non-sterile seawater and air. This likely results in mixed species in the harvested material. Additionally, processing can substantially affect protein structure, digestibility, and allergenicity. Techniques such as fermentation, heating, gamma irradiation, ultrasound, and high-pressure treatments may cause denaturation, aggregation, or epitope modification, potentially increasing or decreasing allergenic potential^50^. Notably, in our study, Ulva-DBF underwent washing and gamma irradiation, which could remove water-soluble molecules, reduce protein solubility and stability, and possibly modify allergenic epitopes^51^. Effects on protein content before and after processing are shown in Fig. 1. Therefore, assessing allergenicity in the final processed product is essential, and further studies are needed to clarify how different processing methods may affect allergenic potential.

It is important to note that, while IgE antibodies in the blood play a central role in allergic responses and are key to various immunological tests, their presence alone is insufficient to predict an allergy. An allergic response upon consumption of the allergen is required for definitive confirmation. Currently, official allergen identification primarily relies on IgE detection (e.g., via ELISA). Diagnostic methods progress in reliability, beginning with IgE diagnosis, moving to the Basophil Activation Test (BAT) (which requires standardized protocols)^52^, then to SPT, and ultimately to OFC, which is considered the most reliable^53^. In Version 22, dated May 25, 2023, the AOL database listed 793 food allergens, 57% of which were identified via IgE binding alone^37,54^, in alignment with the WHO/IUIS Allergen Nomenclature database. Consequently, these allergens are considered putative and require cautious evaluation of suspected protein allergenicity based on homology matches. Moreover, not all researched allergens are likely registered (Table 1); for example, carrot cyclophilin^55^ and Bombay locust GAPDH^36^. In summary, while GAPDH, cyclophilin, and MnSOD share over 70% homology with known food allergens, their allergenicity remains undefined without clinical evidence of allergic reactions triggered by these proteins (Table 1).

Based on our proteomic prevalence calculations (Table 2), the estimated prevalence of allergy to known homology allergens in *Ulva* in the general population is 0.01884% (0.0001884). Assuming this prevalence, the probability of detecting at least one allergic person in a sample of 2000 individuals can be calculated using the formula: P ( ≥ 1 allergic event) = 1−(1−*p*)^*n*^, which is approximately 98%, assuming the calculated prevalence holds true. This demonstrates that the likelihood of detection depends significantly on the sample size and probabilistic thresholds used in the screening. However, this estimate could be lower due to the inclusion of low-abundance proteins, which are less likely to be allergenic compared to more abundant proteins^56^. Nonetheless, given the uncertain thresholds, even small amounts of protein with homologous allergens were considered^57^. For example, pistachio, one of the sources of the homologous proteins detected, can trigger severe allergic reactions with minimal exposure^58^. Additionally, all three homologous allergens identified, based solely on IgE binding (Table 1), might lead to an overestimation of the prevalence of the detected allergens.

The absence of reported allergic reactions does not eliminate the potential risk of Ulva-DBF being allergenic, as this could result from underreporting or limited product use^8^. However, this absence may align with the low allergenic potential emerging from homology-based prevalence calculations, suggesting that detecting a single allergic response would require a study involving thousands of participants. Proteomic analyses are inherently limited, especially for *Ulva* species, due to the lack of an annotated proteome, reliance on incomplete protein databases, and the potential for unidentified, unique proteins at the Ulvophyceae taxonomic level. Consequently, putative allergens can only be identified by similarity to known proteins from other species. Additionally, low-abundance proteins, protein diversity, and post-translational modifications further complicate the interpretation of biological significance, potentially leading to unidentified proteins^59^. Focusing on peptides may improve accuracy^25^. Furthermore, quantification is often relative rather than absolute, and reproducibility can be impacted by sample preparation variability and instrument sensitivity^60^. These limitations emphasize that allergenicity assessments should be conducted within a comprehensive risk management framework. Further limitations stem from the small number of participants in the interim clinical trial, making this a preliminary publication. Nevertheless, documenting these data is essential, as the reported prevalence of discomfort (16.6%) underscores the need for manufacturing adjustments, such as alternative processing methods, if this food is planned for commercialization.

Given these limitations, future research should focus on narrowing the current uncertainty by decoding the *Ulva* genome, identifying its taxa, and studying the safety of its polysaccharides (including ulvan) and proteins^61^, especially as its use in the food industry grows. Moreover, comparing the safety of different *Ulva* species and other green macroalgae, along with testing various clinical trial protocols (e.g., single exposure followed by periods of avoidance before a second exposure), could provide valuable insights into allergenicity. Additionally, this initial allergenicity assessment, which focused on food allergen homology, could be expanded to include contact and airway allergens to uncover potential cross-reactions^26^.

This study represents a significant step in evaluating the allergenicity of Ulva-DBF. A thorough literature search found no documentation of such allergenicity. The currently reported interim clinical trial revealed no allergic reactions following OFC in the studied cohort. However, allergen homology suggests a low allergenic potential (<0.02%)^61^. Accordingly, we envision that Ulva’s potential allergenicity concerns should not hinder its adoption as a sustainable food source. If future evidence identifies allergenicity, regulatory measures such as appropriate labeling can effectively mitigate associated risks, as demonstrated by sesame in the USA and lupin in Australia^8^. Given this context, widespread, long-term consumption in large populations remains the most reliable method for evaluating public health risks. We advocate for the pragmatic integration of novel or less familiar protein sources in the pursuit of innovative and sustainable solutions, while balancing this with post-marketing surveillance to ensure consumer safety.

## Methods

The literature review explored the safe use of marine macroalgae and their potential implications in food allergy cases. Notably, grouping cyanobacteria, microalgae, and macroalgae under the general term “algae” can be misleading for allergenicity assessments, as allergenic cases are common in cyanobacteria but rare in macroalgae^27^. Since *Ulva* is a macroalga, we focused solely on macroalgae (seaweeds). The following databases and tools were utilized: PubMed Central®, Google Scholar, the EFSA journal, and the Elicit AI tool (https://elicit.com/). To explore the possibilities of macroalgal allergens, we also examined related marine realms such as fish and crustaceans (which are common major allergen sources), and green leaves.

The seaweed used in this study belongs to the genus *Ulva* (“sea lettuce”), a green marine macroalga distributed worldwide and found in the intertidal and shallow waters along the Israeli Mediterranean Sea shores. Taxonomic characterization confirmed the species as *Ulva ohnoi*, based on morphological analyses by two experts and genetic analysis at Ghent University (Prof. Olivier De Clerck’s group). Specimens were obtained from stocks cultivated in the outdoor seaweed collection at the Israel Oceanographic and Limnological Research (IOLR) institute in Haifa, Israel. The seaweed was grown in 18 m^3^ PVC U-tanks, 1 m deep, supplied with running seawater, aeration, and weekly fertilization with 0.5 mmol/L NH_4_NO_3_ and 0.04 mmol/L H_3_PO_4_. Water exchange was halted for 24 h after each nutrient application to allow for nutrient uptake. The harvested fresh biomass was rinsed in tap water for 15 min to remove salts, dried at 40 °C until a constant weight was reached, and then milled. This product was named “*Ulva* unwashed” (Ulva-UW). The final product, which is referred to as “Ulva demineralized biomass flour” (Ulva-DBF), was prepared from Ulva-UW as follows: Ulva-UW biomass was washed three times in tap water at a 1:10 dry-weight biomass ratio, dried again at 40 °C until a constant weight was reached, and milled. Ulva-DBF was sterilized using gamma irradiation (Sor-van Ltd, Israel). Approvals for analyses of microbial content, toxins, gluten, pesticides, and iodine are documented in the Supplementary Information.

Two 50 g samples were sent to the Smoler Protein Research Center, Technion, IIT, for proteomic analysis: one containing Ulva-DBF and the other Ulva-UW. The Ulva-DBF sample underwent an additional washing step and gamma irradiation, whereas Ulva-UW remained untreated.

Dry material was treated with SDS buffer (5% SDS, 10 mM DTT, 100 mM TRIS, pH8) and heated (95 °C, 5 min), sonicated twice (90%, for cycles of 10 s on and 10 s off, for 5 min), and centrifuged (10 min, 10,000×*g*, at room temperature). The resulting precipitate was washed 4 times with cold 80% acetone. Protein pellets were dissolved in urea solution (8.5 M urea, 400 mM NH₄HCO₃, 10 mM DTT), and their concentration was determined by Bradford assay. We selected the SDS-based extraction because preliminary urea-only extractions produced smeared chromatograms, likely due to phenolic interference, whereas SDS improved protein solubilization and facilitated efficient downstream proteolysis. The Bradford assay indicated that ~3% of the total biomass was soluble/extractable protein, a typical yield for plant samples and sufficient to ensure reliable downstream proteomic analysis. Samples were reduced with DTT in the urea solution (60 °C, 30 min), then modified with 35.2 mM iodoacetamide in 100 mM NH₄HCO₃ (room temperature, 30 min in the dark) for carbamidomethylation. Subsequently, the samples were digested with modified trypsin (Promega) in urea solution (1.5 M urea, 66 mM NH₄HCO₃) overnight (37 °C, 1:50 enzyme-to-substrate ratio), followed by a second trypsinization step for 4 h (1:100 ratio). Tryptic peptides were desalted using a C18 stage tip (TopTip, Glygen), dried, and re-suspended in 0.1% formic acid.

The resulting peptides were analyzed with LC–MS/MS using a Q Exactive HFX mass spectrometer (Thermo Scientific) coupled with a capillary HPLC system (UltiMate 3000, Thermo Scientific). Peptides were loaded onto a capillary column (30 cm, 75-micron ID) packed with ReproSil C18-Aqua (Dr. Maisch GmbH, Germany) in solvent A (0.1% formic acid in water). A linear gradient of solvent B (99.99% acetonitrile with 0.1% formic acid) resolved the peptide mixture: 5–28% over 180 min, followed by 28–95% over 15 min, held at 95% acetonitrile with 0.1% formic acid for 15 min, all at a flow rate of 0.15 μL/min. Mass spectrometry (MS), in positive mode (*m*/*z* 300–1800), involved full MS scans with a resolution of 120,000 and MS2 scans at 15,000. Full MS scans were followed by high collision dissociation with a normalized collision energy of 27% for the 30 most dominant ions (>1 charge) selected from the first MS scan. Automatic Gain Control settings were 3 × 10^6^ for full MS and 1 × 10^5^ for MS/MS scans, with an intensity threshold of 1 × 10^4^ for triggering MS/MS analysis. Dynamic exclusion was enabled with a 20 s exclusion duration.

MS data were analyzed using MaxQuant software (version 2.1.0.0) with the Andromeda search engine. Searches were performed in the Ulvophyceae section of the National Center for Biotechnology Information (NCBI)-nr database (June 2022, 42,156 entries) with mass tolerances of 6 ppm for precursor masses and 20 ppm for fragment ions. Variable modifications included oxidation on methionine and protein N-terminus acetylation, while carbamidomethylation on cysteine was static. Peptide length was set to a minimum of 7 amino acids, with a maximum of 2 missed cleavages allowed. Label-free analysis was conducted within the same software. Peptide- and protein-level false discovery rates were filtered to 1% using the target-decoy strategy. Identifications from the reverse database and known contaminants, like human keratins and cow serum proteins, were excluded from analysis^62^.

The MS proteomics data were deposited in the ProteomeXchange Consortium via the PRIDE^63^ partner repository with the dataset identifier PXD052629. The dataset is publicly accessible without restrictions.

**Fig. 2:**
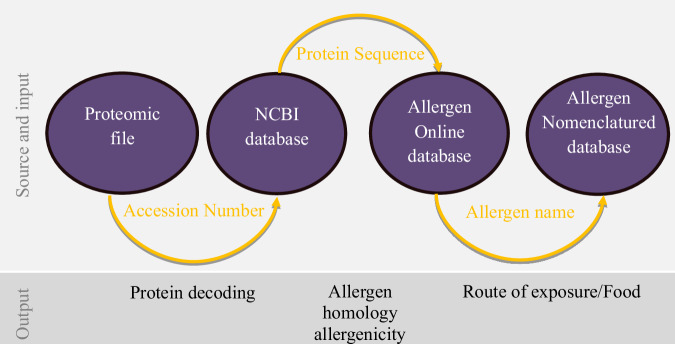
Analyzing protein allergenicity from a proteomics file using online databases. The databases utilized include: **NCBI**: Comprehensive biological information repository (https://www.ncbi.nlm.nih.gov/). **AOL**: Peer-reviewed allergen database for homology searches (http://www.allergenonline.org/). **WHO/IUIS Allergen Nomenclature Database (Allergen.org)**: Source for expert-reviewed allergen data and standardized nomenclature (http://allergen.org). This study utilized the AOL “Sliding 80mer Window 36 search” method^68^ (http://allergenonline.org/databasefasta.shtml, http://www.allergenonline.org/databasehelp.shtml) to compare proteins to allergens in the database. This process generated homology scores for both the Sliding 80mer Window and full-alignment sequence, alongside allergen names. Our output was organized by biochemical and allergen names, with duplicates removed and food allergens consolidated if their sliding 80mer window sequences exceeded 35%.

Regulatory requirements suggest that sequences demonstrating at least 35% 80mer sliding window homology are deemed “likely to be allergenic”^8^. However, research indicates that an identity threshold exceeding 50% detects potential weak cross-reactivity, while levels above 70% typically indicate high cross-reactivity potential^64^. Additionally, whole protein sequence analysis has been shown to reduce false positives while maintaining equivalent rates of false negatives compared to the sliding-window approach^65,66^. Therefore, our analysis presented both 80mer sliding window homology and whole protein sequence homology. We specifically focused on proteins with homology results above 70% for food allergens. Figure 2 depicts the data-gathering process. Similar protocols were followed in previous studies^26,67^.

Intensity-Based Absolute Quantitation (iBAQ) assesses relative protein abundance by summing intensity values, which collectively represent the total protein amount. The relative abundance of a specific protein is calculated as (P_iBAQ/∑P_iBAQ × 100), where P_iBAQ is the iBAQ value of the protein and ∑P_iBAQ is the total of iBAQ protein values in the sample.

This study reports the pilot phase of a clinical trial involving 31 participants. Healthy adults with no history of atopy were recruited from Tel Aviv University and Meir Medical Center staff and students from October 2024 to July 2025. Baseline demographic characteristics of the participants are summarized in Table 3. The mean age and standard deviation were 43.0 ± 10.8 years, and 17 participants (54.8%) were female. They received a brochure outlining the study requirements. Participants were asked to consume 15 g of Ulva-DBF (equivalent to 5 g of protein) twice a week for six weeks. Before the feeding phase, volunteers underwent SPT with Ulva-DBF extract and common allergens, including fish, seafood, peanuts, sesame, milk, and soy. Individuals sensitized to these allergens were excluded. Participants consumed Ulva-DBF under the direct supervision of an allergist at the study center, incorporating the flour into water or common food items from their menu. After one month of avoidance, they underwent another SPT and an open oral Ulva-DBF challenge. Allergic reactions were assessed by the allergist based on established clinical signs and symptoms.

**Table 3:** Baseline characteristics of participants

Characteristic	All participants (*N* = 31)
Age (years), mean ± SD	43.0 ± 10.8
Female, *n* (%)	17 (54.8%)
Male, *n* (%)	14 (45.2%)
History of atopy	Excluded from recruitment

This clinical trial was conducted in accordance with the World Medical Association’s Declaration of Helsinki, approved by the Meir Medical Center Ethics Committee (IRB number: MMC-0207-21), and registered at ClinicalTrials.gov (Identifier: NCT06452381; first posted date: June 11, 2024). Written informed consent was obtained from all participants prior to enrollment. A completed CONSORT 2010 extension checklist for randomized pilot and feasibility trials (Eldridge et al., BMJ 2016) is provided in the Supplementary Information.

## Supplementary information

Supplementary Information

Supplementary Data1

## Data Availability

The data that support the findings of this study are available from the corresponding authors upon reasonable request.
