# Supplementary material for: Initial allergenicity assessment of Ulva sp. seaweed flour
Source: NPJ Sci Food. 2025 Dec 3;10:1. doi: 10.1038/s41538-025-00638-x (PMC12764789; doi:10.1038/s41538-025-00638-x)
Supplement: Supplementary file 1 — Supplementary Information [file 41538_2025_638_MOESM1_ESM.pdf]

Supplementary Materials: Language Note

Title of Manuscript: Initial Allergenicity Assessment of Ulva sp. Seaweed Flour

Please note that the original laboratory results and certain supplementary documents are in Hebrew. For the purpose of submission and review, we have provided accurate English translations of these documents.

The English versions should be considered the official documents for evaluation. The original Hebrew files are available upon request for verification or regulatory purposes.

This note is included to ensure full transparency and to comply with the requirement that all supplementary materials be provided in English.

Aminolab Ltd. Analytical Laboratory Services
T +972.8.930.3333 F +972.8.930.3300
Pinhas Sapir St., Kiryat Weizmann 1
P.O. Box 4074 Nes Ziona, 70400

To: Efraim Steinbruch
Tel Aviv University
Email: efraim.gs@gmail.com

Laboratory Results Report: Certificate No. 047547.21

Date: 13/12/2021

Aminolab No.: 099563.21-CMP

Sample description: UL81121 – Algae Powder

Date received: 17/11/2021

Sampled by: The client

Order No.: 35 7265710

Test Results:

Test	Result	Units	Notes
Ash	17	%	(-)
Protein	35	%	1
Mercury - Cold Vapor	<0.1	mg/kg	(-)
ICP - Metal Scan			2
Arsenic - As	1	mg/kg	-
Cadmium – Cd	0.2	mg/kg	-
Lead - Pb	0.3	mg/kg	-

Test comments:

No comments = (-)

1. Protein - Factor for protein calculation: 6.25
2. ICP - Metal Scan - results including "<" = below reporting limits

Quality Assurance:

Test	Standard / Method	Units
Ash	Based on AOAC 920.153	A
Protein	Based on AOAC 981.10	A
Mercury - Cold Vapor	Based on EPA 7473	(-)
ICP - Metal Scan	SOP# 20.S.111	(-)

Approved by: Department Head – Dr. Irina Rubinstein

The information provided reflects the exact results of the sample submitted for testing as received by the laboratory. The name or reputation of Aminolab Ltd. may not be used in connection with the data or findings specified in this document except with its prior written permission. This document and the data therein must be referenced in full and may not be copied or quoted, in whole or in part, in other documents.

Aminolab Ltd. Analytical Laboratory Services
T +972.8.930.3333 F +972.8.930.3300

Certificate No.: 047547.21
Aminolab No.: 099563.21-CMP
Date: 13/12/2021

The laboratory's quality system is certified according to ISO/IEC 17025 and operates under regulated work procedures.

Accreditation / Recognition:
A. The laboratory is accredited for this test according to ISO/IEC 17025 by the National Authority for Laboratory Accreditation.
The National Authority for Laboratory Accreditation is not responsible for the test results issued by the laboratory, and the accreditation does not constitute approval of the tested item.
B. [If not accredited] = no accreditation or recognition.

Approved by:
Department Head – Dr. Irina Rubinstein

The information provided reflects the exact results of the sample submitted for testing as received by the laboratory. The name or reputation of Aminolab Ltd. may not be used in connection with the data or findings specified in this document except with its prior written permission. This document and the data therein must be referenced in full and may not be copied or quoted, in whole or in part, in other documents.

- End of test certificate

Aminolab Ltd. Analytical Laboratory Services
T +972.8.930.3333 F +972.8.930.3300
Pinhas Sapir St., Kiryat Weizmann 1
P.O. Box 4074 Nes Ziona, 70400

To: Dr. Alex Goldberg
Tel Aviv University
Email: agolberg@gmail.com
Copy: julia.wise905@gmail.com;

Laboratory Results Report: Certificate No. 024925.22

Date: 24/07/2022

Aminolab No.: 054711.22-M

Sample description: Dried seaweed, Ulva type

Date received: 06/07/2022

Sampled by: The client

Order No.: 72-7326744

Test Start: 10/07/2022

Test End: 13/07/2022

Storage temperature: Room temperature

Test Results:

Test	Result	Units	Notes
Total count	5000	CFU/gr	(-)
Coliforms	<10	CFU/gr	(-)
Staphylococcus aureus coagulase-positive	<50	CFU/gr	(-)
Yeasts / Molds<br>Yeasts<br>Molds	 <10<br><10	 CFU/gr<br>CFU/gr	(-)<br>-<br>-
Salmonella	Negative	25gr	**1**
Escherichia coli (beta method, CFU/gr)	<10	CFU/gr	(-)
Gluten (ppm)	<5	ppm	**2**

Test comments:

No comments = (-)

1. Salmonella - Negative result - No presence detected in 25g sample
2. Permitted gluten level <20ppm as per 2011 Public Health Regulations (Gluten marking amendment)
3. Approved by: Department Head – Dr. Helena Ashkenazi

The information detailed accurately reflects the results of the sample as received by the laboratory. The name or reputation of Aminolab Ltd. may not be used in relation to the data or findings in this document except with prior written approval. The data in this document must be referenced in full and must not be copied or cited, entirely or in part, in other documents.

Aminolab Ltd. Analytical Laboratory Services
T +972.8.930.3333 F +972.8.930.3300

Certificate No.: 024925.22
Aminolab No.: 054711.22-M
Date: 24/07/2022

Quality Assurance:

**Test**	**Standard / Method**
Total count	IS 885 part 3
Coliforms	IS 885 part 4
Staphylococcus aureus coagulase-positive	IS 885 part 6
Yeasts / Molds	IS 885 part 8
Salmonella	IS 885 part 7, ISO 6579
Escherichia coli (beta method)	IS 885 part 12
Gluten	ELISA Enzyme immunoassay

Accreditation / Recognition:

The laboratory's quality system is certified according to ISO/IEC 17025 and operates under regulated work procedures.

A. The laboratory is recognized for conducting this test by the Ministry of Health.

B. Accredited for this test under ISO/IEC 17025 by the National Laboratory Accreditation Authority.

The Accreditation Authority is not responsible for test results produced by the laboratory, and accreditation/recognition does not constitute approval of the item, system, or process tested. Reference must be made to the complete document, and copying sections into other documents is not permitted. Authority accreditation does not validate laboratory procedures or staff.

Approved by:Department Head – Dr. Helena Ashkenazi

The information detailed reflects the results of the submitted sample as received by the laboratory. Use of Aminolab Ltd.'s name or reputation in connection with these data or findings is not permitted except with prior written consent. Reference must be made to the full document, and no part may be copied or cited in other documents.

- End of test certificate

Aminolab Ltd. Analytical Laboratory Services
T +972.8.930.3333 F +972.8.930.3300
Pinhas Sapir St., Kiryat Weizmann 1
P.O. Box 4074 Nes Ziona, 70400

To: Efraim Steinbruch
Tel Aviv University
Email: efraim.gs@gmail.com

Laboratory Results Report: Certificate No. **046082.21**

Date: 02/12/2021

Aminolab No.: 099563.21-CMP

Sample description: UL81121 - Algae Powder

Date received: 17/11/2021

Sampled by: Client

Order No.: 35 7265710

Test Results:

Test	Units	Result	Notes
**Aflatoxins G1 G2 B1 B2**			1
G1 Aflatoxin	ppb	ND	-
G2 Aflatoxin	ppb	ND	-
B1 Aflatoxin	ppb	ND	-
B2 Aflatoxin	ppb	ND	-
**Ochratoxin A**	ppb	ND	2

ND (Not Detected): Below the detection limit.

Key Notes:

1. **Aflatoxins:** Total permissible aflatoxin levels (B1 included) in food in Israel: 5ppb, total **Aflatoxins** – 15ppb.

2. **Ochratoxin A:** Permissible level for grains, legumes, and their products in Israel: 50 ppb.

Quality Assurance:

Test	Testing Methods and Standards	Certification/Accreditation
**Aflatoxins G1 G2 B1 B2**	Based on AOAC 994.08 + 999.07, by HPLC method with immunoaffinity clean up. LOD: for Aflatoxins B2,G1,G2 = 1ppb, for Aflatoxin B1=0.5ppb	A, B
**Ochratoxin A**	Based on AOAC 991.44, by HPLC, LOD=0.5ppb.	A

Approved By: Dr. Yoram Cohen - Department Manager

The information detailed accurately reflects the results of the sample as received by the laboratory. The name or reputation of Aminolab Ltd. may not be used in relation to the data or findings in this document except with prior written approval. The data in this document must be referenced in full and must not be copied or cited, entirely or in part, in other documents.

Accreditation / Recognition:

The laboratory's quality system is certified according to ISO/IEC 17025 and operates under regulated work procedures.

A. The laboratory is recognized for conducting this test by the Ministry of Health.

B. Accredited for this test under ISO/IEC 17025 by the National Laboratory Accreditation Authority.

The Accreditation Authority is not responsible for test results produced by the laboratory, and accreditation/recognition does not constitute approval of the item, system, or process tested. Reference must be made to the complete document, and copying sections into other documents is not permitted. Authority accreditation does not validate laboratory procedures or staff.

Approved by:Department Head – Dr. Helena Ashkenazi

The information detailed reflects the results of the submitted sample as received by the laboratory. Use of Aminolab Ltd.'s name or reputation in connection with these data or findings is not permitted except with prior written consent. Reference must be made to the full document, and no part may be copied or cited in other documents.

- End of test certificate

Aminolab Ltd. Analytical Laboratory Services
T +972.8.930.3333 F +972.8.930.3300
Pinhas Sapir St., Kiryat Weizmann 1
P.O. Box 4074 Nes Ziona, 70400

To: Efraim Steinbruch
Tel Aviv University
Email: efraim.gs@gmail.com

Laboratory Results Report: Certificate No. **046082.21**

Date: 02/12/2021

Aminolab No.: 099563.21-CMP

Sample description: UL81121 - Algae Powder

Date received: 17/11/2021

Sampled by: Client

Order No.: 35 7265710

Test Results:

Test	Unit	Result	Comments
**Pesticide Residue by GC-MS**	**mg/Kg**	**Not Detected**	**1**
**Pesticide Residue by LC-MS/MS**	**mg/Kg**	**Not Detected**	**1**

Test Comments:

No comments (--)

1. A list of tested substances, including detection and quantification limits and certification, was sent to the client.

Quality Assurance:

Test	Testing Methods and Standards	Certification/ Accreditation
**Pesticide Residue by GC-MS**	In-house procedure by GC-MS	A, B
**Pesticide Residue by LC-MS/MS**	In-house procedure by LC-MS/MS	A, B

Accreditation / Recognition:

The laboratory's quality system is certified according to ISO/IEC 17025 and operates under regulated work procedures.

A. The laboratory is recognized for conducting this test by the Ministry of Health.

B. Accredited for this test under ISO/IEC 17025 by the National Laboratory Accreditation Authority. The Accreditation Authority is not responsible for test results produced by the laboratory, and accreditation/recognition does not constitute approval of the item, system, or process tested.

Approved By: Dr. Yoram Cohen - Department Manager

The information detailed accurately reflects the results of the sample as received by the laboratory. The name or reputation of Aminolab Ltd. may not be used in relation to the data or findings in this document except with prior written approval. The data in this document must be referenced in full and must not be copied or cited, entirely or in part, in other documents.

BACTOCHEM בקטוכם
An Emilia Development Company

Tel: 08-9308308
Fax: 08-9300991, 08-9401439
18 Hacharash st., Ness Ziona

service@bactochem.co.il
www.bactochem.co.il

Test Certificate No.: 920380

Final Report

Contact Person		Client Details	
Name:	Mobile:	Name: Mor Application Research	City: Ramat Gan
Phone:	Fax:	Address:	Postal Code:

Work Order No.: D090622-0045

Sampling Form No.: 09/06/2022 (client's sampling form) 12:05:00

Sampler: By client

Sample Description: Ulva seaweed **Sample No.:** 1386284

Sample Storage and Transport Conditions: Refrigerated Sampling Date: 09/06/2022

Test	Method	*LOQ	Result	Permissible Range	Unit	Notes
Iodine (I2)	AOAC 935.14	10	125.0		mg/kg	–

Notes

- The results refer only to the tested item.
- The reference for the "permissible range" values is indicated as a note.
- This document must be considered in full and may not be copied into other documents.
- The laboratory's measurement standards are calibrated in accredited laboratories in accordance with ISO/IEC 17025 and are traceable to national or international standards.
- LOQ = MRL: The limit of quantification of the test method.
- The reported results do not include uncertainty values; therefore, compliance with specification standards cannot be determined.
- This document is provided for the exclusive use of the recipient client. The document, the company's name, or the name of any of its employees may not be used for advertising or sales purposes without prior written approval from Bactochem Laboratories Ltd.
- Bactochem Laboratory is accredited by the Israel Laboratory Accreditation Authority (ISRAC) and operates in accordance with the requirements of ISO/IEC 17025, within the scope of accreditation detailed in the annex to the accreditation certificate.
- Use of the ISRAC symbol refers only to tests that are within the organization's scope of accreditation and are performed in accordance with accreditation rules as specified in the accreditation certificate.
- The Israel Laboratory Accreditation Authority is not responsible for the results of the tests performed by the laboratory, and accreditation does not constitute approval of the tested item.
- Opinions or interpretations are not covered under the accreditation of the Israel Laboratory Accreditation Authority.
- Tests marked (1) are accredited by the Israel Laboratory Accreditation Authority.

The results in this certificate have been approved by:

Lush Cernes, Food Chemistry and Pesticide Departments Manager

— End of Certificate —

Supplementary Data 1. Reference

1. Ruethers T, Taki AC, Karnaneedi S, et al. Expanding the allergen repertoire of salmon and catfish. *Allergy*. 2021;76(5):1443-1453. doi:https://doi.org/10.1111/all.14574

2. Wang S, Chen H, Tang X, et al. The Role of Glyceraldehyde-3-Phosphate Dehydrogenases in NADPH Supply in the Oleaginous Filamentous Fungus Mortierella alpina. *Front Microbiol*. 2020;11. doi:10.3389/FMICB.2020.00818/FULL

3. Mattsson L, Valcour A, Holmqvist M, Larsson H, Lidholm J. Cyclophilin – A novel cross-reactive determinant in peanut. *Clinical & Experimental Allergy*. 2021;51(4):620-622. doi:10.1111/CEA.13833

4. Flückiger S, Fijten H, Whitley P, Blaser K, Crameri R. Cyclophilins, a new family of cross-reactive allergens. *Eur J Immunol*. 2002;32(1):10-17. doi:10.1002/1521-4141

5. Wang P, Heitman J. The cyclophilins. *Genome Biol*. 2005;6(7):1-6. doi:10.1186/GB-2005-6-7-226/FIGURES/3

6. Li Y, Sun X, Yang L. Structure/epitope analysis and IgE binding activities of three cyclophilin family proteins from Dermatophagoides pteronyssinus. *Scientific Reports 2023 13:1*. 2023;13(1):1-10. doi:10.1038/s41598-023-40720-6

7. Kuehn A, Hilger C, Lehners-Weber C, et al. Identification of enolases and aldolases as important fish allergens in cod, salmon and tuna: component resolved diagnosis using parvalbumin and the new allergens. *Clinical & Experimental Allergy*. 2013;43(7):811-822. doi:10.1111/cea.12117

8. Morales-Amparano MB, Huerta-Ocampo JÁ, Pastor-Palacios G, Teran LM. The Role of Enolases in Allergic Disease. *Journal of Allergy and Clinical Immunology: In Practice*. 2021;9(8):3026-3032. doi:10.1016/j.jaip.2021.04.005

9. Kuehn A, Codreanu-Morel F, Lehners-Weber C, et al. Cross-reactivity to fish and chicken meat - a new clinical syndrome. *Allergy*. 2016;71(12):1772-1781. doi:10.1111/ALL.12968

10. Noorbakhsh R, Mortazavi SA, Sankian M, Shahidi F, Assarehzadegan MA, Varasteh AR. Cloning, expression, characterization, and computational approach for cross-reactivity prediction of manganese superoxide dismutase allergen from pistachio nut. *Allergol Int*. 2010;59(3):295-304. doi:10.2332/ALLERGOLINT.10-OA-0174

11. Costa J, Silva I, Vicente AA, Oliveira MBPP, Mafra I. Pistachio nut allergy: An updated overview. *Crit Rev Food Sci Nutr*. 2019;59(4):546-562. doi:10.1080/10408398.2017.1379947

12. Davis CA, Hearn AS, Fletcher B, et al. Potent Anti-tumor Effects of an Active Site Mutant of Human Manganese-Superoxide Dismutase. *Journal of Biological Chemistry*. 2004;279(13):12769-12776. doi:10.1074/jbc.m310623200

13. Kwak SE, Shin HE, Zhang D Di, et al. Potential role of exercise-induced glucose-6-phosphate isomerase in skeletal muscle function. *J Exerc Nutrition Biochem*. 2019;23(2):28-33. doi:10.20463/JENB.2019.0014

14. Pirovich DB, Da'dara AA, Skelly PJ. Multifunctional Fructose 1,6-Bisphosphate Aldolase as a Therapeutic Target. *Front Mol Biosci*. 2021;8:719678. doi:10.3389/FMOLB.2021.719678/BIBTEX

15. Wai CYY, Leung NYH, Leung ASY, et al. Comprehending the allergen repertoire of shrimp for precision molecular diagnosis of shrimp allergy. *Allergy*. 2022;77(10):3041-3051. doi:10.1111/ALL.15370

16. Hudson JW, Golding GB, Crerar MM. Evolution of allosteric control in glycogen phosphorylase. *J Mol Biol*. 1993;234(3):700-721. doi:10.1006/JMBI.1993.1621

17. Alonso-Casajús N, Dauvillée D, Viale AM, et al. Glycogen phosphorylase, the product of the glgP gene, catalyzes glycogen breakdown by removing glucose

units from the nonreducing ends in Escherichia coli. *J Bacteriol*. 2006;188(14):5266-5272. doi:10.1128/JB.01566-05

18. Khanaruksombat S, Srisomsap C, Chokchaichamnankit D, Punyarit P, Phiriyangkul P. Identification of a novel allergen from muscle and various organs in banana shrimp (Fenneropenaeus merguiensis). *Annals of Allergy, Asthma and Immunology*. 2014;113(3):301-306. doi:10.1016/j.anai.2014.06.002

19. Grijincu M, Huțu I, Weber M, et al. Physicochemical and immunological characterization of Amb a 12, a novel ragweed (Ambrosia artemisiifolia) pollen allergen. *Mol Immunol*. 2023;157:18-29. doi:10.1016/J.MOLIMM.2023.03.012

20. Klug C, Hemmer W, Román-Carrasco P, et al. Gal d 7—a major allergen in primary chicken meat allergy. *Journal of Allergy and Clinical Immunology*. 2020;146(1):169-179.e5. doi:10.1016/j.jaci.2020.02.033

21. Heissler SM, Sellers JR. Myosin light chains: Teaching old dogs new tricks. *Bioarchitecture*. 2014;4(6):169-188. doi:10.1080/19490992.2015.1054092

22. Zhang YX, Chen HL, Maleki SJ, et al. Purification, Characterization, and Analysis of the Allergenic Properties of Myosin Light Chain in Procambarus clarkii. *J Agric Food Chem*. 2015;63(27):6271-6282. doi:10.1021/ACS.JAFC.5B01318

23. Bauermeister K, Wangorsch A, Garoffo LP, et al. Generation of a comprehensive panel of crustacean allergens from the North Sea Shrimp Crangon crangon. *Mol Immunol*. 2011;48(15-16):1983-1992. doi:10.1016/J.MOLIMM.2011.06.216

24. Li MS, Xia F, Liu M, et al. Cloning, Expression, and Epitope Identification of Myosin Light Chain 1: An Allergen in Mud Crab. *J Agric Food Chem*. 2019;67(37):10458-10469. doi:10.1021/ACS.JAFC.9B04294

25. Ayuso R, Grishina G, Bardina L, et al. Myosin light chain is a novel shrimp allergen, Lit v 3. *J Allergy Clin Immunol*. 2008;122(4):795-802. doi:10.1016/J.JACI.2008.07.023

26. Kalic T, Kamath SD, Ruethers T, et al. Collagen-An Important Fish Allergen for Improved Diagnosis. *J Allergy Clin Immunol Pract*. 2020;8(9):3084-3092.e10. doi:10.1016/J.JAIP.2020.04.063

27. Kobayashi Y, Akiyama H, Huge J, et al. Fish collagen is an important panallergen in the Japanese population. *Allergy*. 2016;71(5):720-723. doi:10.1111/ALL.12836

28. Haq F, Ahmed N, Qasim M. Comparative genomic analysis of collagen gene diversity. *3 Biotech*. 2019;9(3):1-9. doi:10.1007/S13205-019-1616-9/FIGURES/4

29. Gillis TE, Marshall CR, Tibbits GF. Functional and evolutionary relationships of troponin C. *Physiol Genomics*. 2007;32(1):16-27. doi:10.1152/PHYSIOLGENOMICS.00197.2007

30. Yang AC, Arruda LK, Santos ABR, et al. Measurement of IgE antibodies to shrimp tropomyosin is superior to skin prick testing with commercial extract and measurement of IgE to shrimp for predicting clinically relevant allergic reactions after shrimp ingestion. *J Allergy Clin Immunol*. 2010;125(4):872-878. doi:10.1016/J.JACI.2009.11.043

31. Papia F, Bellia C, Uasuf CG. Tropomyosin: A panallergen that causes a worldwide allergic problem. *Allergy Asthma Proc*. 2021;42(5):E145-E151. doi:10.2500/AAP.2021.42.210057

32. Kamath SD, Bublin M, Kitamura K, Matsui T, Ito K, Lopata AL. Cross-reactive epitopes and their role in food allergy. *Journal of Allergy and Clinical Immunology*. 2023;151:1178-1190. doi:10.1016/j.jaci.2022.12.827

33. Yu R, Ono S. Dual roles of tropomyosin as an F-actin stabilizer and a regulator of muscle contraction in Caenorhabditis elegans body wall muscle. *Cell Motil Cytoskeleton*. 2006;63(11):659-672. doi:10.1002/CM.20152

34. Ahumada V, García E, Dennis R, et al. IgE responses to Ascaris and mite tropomyosins are risk factors for asthma. *Clinical & Experimental Allergy*. 2015;45(7):1189-1200. doi:10.1111/CEA.12513

35. Miyazawa H, Fukamachi H, Inagaki Y, et al. Identification of the first major allergen of a squid (Todarodes pacificus). *J Allergy Clin Immunol*. 1996;98(5 Pt 1):948-953. doi:10.1016/S0091-6749(96)80011-X

36. Asturias JA, Eraso E, Moneo I, Martinez A. Is tropomyosin an allergen in Anisakis? *Allergy*. 2000;55(9):898-898. doi:10.1034/J.1398-9995.2000.00734.X

37. Weichel M, Glaser AG, Ballmer-Weber BK, Schmid-Grendelmeier P, Crameri R. Wheat and maize thioredoxins: a novel cross-reactive cereal allergen family related to baker's asthma. *J Allergy Clin Immunol*. 2006;117(3):676-681. doi:10.1016/J.JACI.2005.11.040

38. Limacher A, Glaser AG, Meier C, et al. Cross-Reactivity and 1.4-Å Crystal Structure of Malassezia sympodialis Thioredoxin (Mala s 13), a Member of a New Pan-Allergen Family. *The Journal of Immunology*. 2007;178(1):389-396. doi:10.4049/JIMMUNOL.178.1.389

39. AlOkda A, Van Raamsdonk JM. Evolutionarily Conserved Role of Thioredoxin Systems in Determining Longevity. *Antioxidants*. 2023;12(4):944. doi:10.3390/ANTIOX12040944/S1

40. Carballeda-Sangiao N, Rodríguez-Mahillo AI, Careche M, et al. Ani s 11-Like Protein Is a Pepsin- and Heat-Resistant Major Allergen of Anisakis spp. and a Valuable Tool for Anisakis Allergy Component-Resolved Diagnosis. *Int Arch Allergy Immunol*. 2016;169(2):108-112. doi:10.1159/000444981

41. Sanchez-Monge R, Lopez-Torrejón G, Pascual CY, Varela J, Martin-Esteban M, Salcedo G. Vicilin and convicilin are potential major allergens from pea. *Clinical & Experimental Allergy*. 2004;34(11):1747-1753. doi:10.1111/J.1365-2222.2004.02085.X

42. Cruciani V, Mikalsen SO. Evolutionary selection pressure and family relationships among connexin genes. *Biol Chem*. 2007;388(3):253-264. doi:10.1515/BC.2007.028

43. Croy RR, Gatehouse JA, Tyler M, Boulter D. The purification and characterization of a third storage protein (convicilin) from the seeds of pea

(Pisum sativum L.). *Biochemical Journal*. 1980;191(2):509-516. doi:10.1042/BJ1910509

44. Rodriguez-Perez R, Moneo I, Rodriguez-Mahillo A, Caballero ML. Cloning and expression of Ani s 9, a new Anisakis simplex allergen. *Mol Biochem Parasitol*. 2008;159(2):92-97. doi:10.1016/J.MOLBIOPARA.2008.02.008

45. Andrés O, Kellermann T, López-Giráldez F, Rozas J, Domingo-Roura X, Bosch M. RPS4Y gene family evolution in primates. *BMC Evol Biol*. 2008;8(1):1-12. doi:10.1186/1471-2148-8-142/TABLES/4

46. Mushtaq M, Ali RH, Kashuba V, et al. S18 family of mitochondrial ribosomal proteins: evolutionary history and Gly132 polymorphism in colon carcinoma. *Oncotarget*. 2016;7(34):55649-55662. doi:10.18632/ONCOTARGET.10957

47. Palacín A, Tordesillas L, Gamboa P, et al. Characterization of peach thaumatin-like proteins and their identification as major peach allergens. *Clinical & Experimental Allergy*. 2010;40(9):1422-1430. doi:10.1111/J.1365-2222.2010.03578.X

48. Hauser M, Roulias A, Ferreira F, Egger M. Panallergens and their impact on the allergic patient. *Allergy Asthma Clin Immunol*. 2010;6(1):1. doi:10.1186/1710-1492-6-1

49. de Jesús-Pires C, Ferreira-Neto JRC, Pacifico Bezerra-Neto J, et al. Plant Thaumatin-like Proteins: Function, Evolution and Biotechnological Applications. *Curr Protein Pept Sci*. 2019;21(1):36-51. doi:10.2174/1389203720666190318164905

50. Pérez-Pérez J, Fernández-Caldas E, Marañón F, et al. Molecular cloning of paramyosin, a new allergen of Anisakis simplex. *Int Arch Allergy Immunol*. 2000;123(2):120-129. doi:10.1159/000024442

51. Ruethers T, Taki AC, Johnston EB, et al. Seafood allergy: A comprehensive review of fish and shellfish allergens. *Mol Immunol*. 2018;100:28-57. doi:10.1016/J.MOLIMM.2018.04.008

52. Yang Y, Zhao D, Zhou L, et al. Molecular Characterization and Expression Pattern of Paramyosin in Larvae and Adults of Yesso Scallop. *Biology 2022, Vol 11, Page 453*. 2022;11(3):453. doi:10.3390/BIOLOGY11030453

53. Yu C, Gao X, Lin H, et al. Purification, Characterization, and Three-Dimensional Structure Prediction of Paramyosin, a Novel Allergen of Rapana venosa. *J Agric Food Chem*. 2020;68(49):14632-14642. doi:10.1021/ACS.JAFC.0C04418

54. Suzuki M, Kobayashi Y, Hiraki Y, Nakata H, Shiomi K. Paramyosin of the disc abalone Haliotis discus discus: Identification as a new allergen and cross-reactivity with tropomyosin. *Food Chem*. 2011;124(3):921-926. doi:10.1016/J.FOODCHEM.2010.07.020

55. Mittag D, Akkerdaas J, Ballmer-Weber BK, et al. Ara h 8, a Bet v 1-homologous allergen from peanut, is a major allergen in patients with combined birch pollen and peanut allergy. *Journal of Allergy and Clinical Immunology*. 2004;114(6):1410-1417. doi:10.1016/j.jaci.2004.09.014

56. Xu TF, Zhao XC, Jiao YT, Wei JY, Wang L, Xu Y. A Pathogenesis Related Protein, VpPR-10.1, from Vitis pseudoreticulata: An Insight of Its Mode of Antifungal Activity. *PLoS One*. 2014;9(4):e95102. doi:10.1371/JOURNAL.PONE.0095102

57. Griesmeier U, Vázquez-Cortés S, Bublin M, et al. Expression levels of parvalbumins determine allergenicity of fish species. *Allergy*. 2010;65(2):191-198. doi:10.1111/J.1398-9995.2009.02162.X

58. Lee P, Nordlee JA, Koppelman SJ, Baumert JL, Taylor SL. Characterization of IgG and IgE Binding to Parvalbumin Derived from Commercially Important Fish Species. *Journal of Allergy and Clinical Immunology*. 2011;127(2):AB32-AB32. doi:10.1016/j.jaci.2010.12.137

59. Wang AS, Wan X, Storch DS, et al. Cross-species conservation in the regulation of parvalbumin by perineuronal nets. *Front Neural Circuits*. 2023;17:1297643. doi:10.3389/FNCIR.2023.1297643/BIBTEX

60. Hänninen AR, Mikkola JH, Kalkkinen N, et al. Increased allergen production in turnip (Brassica rapa) by treatments activating defense mechanisms. *J Allergy Clin Immunol*. 1999;104(1):194-201. doi:10.1016/S0091-6749(99)70135-1

61. Berthelot K, Peruch F, Lecomte S. Highlights on Hevea brasiliensis (pro)hevein proteins. *Biochimie*. 2016;127:258-270. doi:10.1016/J.BIOCHI.2016.06.006

62. Lauer I, Foetisch K, Kolarich D, et al. Hazelnut (Corylus avellana) vicilin Cor a 11: molecular characterization of a glycoprotein and its allergenic activity. *Biochemical Journal*. 2004;383(2):327-334. doi:10.1042/BJ20041062

63. Witke W. The role of profilin complexes in cell motility and other cellular processes. *Trends Cell Biol*. 2004;14(8):461-469. doi:10.1016/j.tcb.2004.07.003

CONSORT 2010 checklist of information to include when reporting a pilot or feasibility trial*

Section/Topic	Item No	Checklist item	Reported on page No
**Title and abstract**			
	1a	Identification as a pilot or feasibility randomised trial in the title	2
	1b	Structured summary of pilot trial design, methods, results, and conclusions (for specific guidance see CONSORT abstract extension for pilot trials)	2
**Introduction**			
Background and objectives	2a	Scientific background and explanation of rationale for future definitive trial, and reasons for randomised pilot trial	3
	2b	Specific objectives or research questions for pilot trial	3
**Methods**			
Trial design	3a	Description of pilot trial design (such as parallel, factorial) including allocation ratio	14
	3b	Important changes to methods after pilot trial commencement (such as eligibility criteria), with reasons	14
Participants	4a	Eligibility criteria for participants	14
	4b	Settings and locations where the data were collected	14
	4c	How participants were identified and consented	14
Interventions	5	The interventions for each group with sufficient details to allow replication, including how and when they were actually administered	14
Outcomes	6a	Completely defined prespecified assessments or measurements to address each pilot trial objective specified in 2b, including how and when they were assessed	14
	6b	Any changes to pilot trial assessments or measurements after the pilot trial commenced, with reasons	14
	6c	If applicable, prespecified criteria used to judge whether, or how, to proceed with future definitive trial	
Sample size	7a	Rationale for numbers in the pilot trial	14
	7b	When applicable, explanation of any interim analyses and stopping guidelines	
Randomisation:			N/A
Sequence generation	8a	Method used to generate the random allocation sequence	N/A
	8b	Type of randomisation(s); details of any restriction (such as blocking and block size)	N/A
Allocation concealment	9	Mechanism used to implement the random allocation sequence (such as sequentially numbered containers), describing any steps taken to conceal the sequence until interventions were assigned	N/A

mechanism			
Implementation	10	Who generated the random allocation sequence, who enrolled participants, and who assigned participants to interventions	N/A
Blinding	11a	If done, who was blinded after assignment to interventions (for example, participants, care providers, those assessing outcomes) and how	N/A
	11b	If relevant, description of the similarity of interventions	N/A
Statistical methods	12	Methods used to address each pilot trial objective whether qualitative or quantitative	N/A
**Results**			
Participant flow (a diagram is strongly recommended)	13a	For each group, the numbers of participants who were approached and/or assessed for eligibility, randomly assigned, received intended treatment, and were assessed for each objective	6
	13b	For each group, losses and exclusions after randomisation, together with reasons	6
Recruitment	14a	Dates defining the periods of recruitment and follow-up	6
	14b	Why the pilot trial ended or was stopped	6
Baseline data	15	A table showing baseline demographic and clinical characteristics for each group	6
Numbers analysed	16	For each objective, number of participants (denominator) included in each analysis. If relevant, these numbers should be by randomised group	N/A
Outcomes and estimation	17	For each objective, results including expressions of uncertainty (such as 95% confidence interval) for any estimates. If relevant, these results should be by randomised group	N/A
Ancillary analyses	18	Results of any other analyses performed that could be used to inform the future definitive trial	6
Harms	19	All important harms or unintended effects in each group (for specific guidance see CONSORT for harms)	6
	19a	If relevant, other important unintended consequences	N/A
**Discussion**			
Limitations	20	Pilot trial limitations, addressing sources of potential bias and remaining uncertainty about feasibility	9
Generalisability	21	Generalisability (applicability) of pilot trial methods and findings to future definitive trial and other studies	9
Interpretation	22	Interpretation consistent with pilot trial objectives and findings, balancing potential benefits and harms, and considering other relevant evidence	9
	22a	Implications for progression from pilot to future definitive trial, including any proposed amendments	9
**Other information**			
Registration	23	Registration number for pilot trial and name of trial registry	14
Protocol	24	Where the pilot trial protocol can be accessed, if available	14
Funding	25	Sources of funding and other support (such as supply of drugs), role of funders	15
	26	Ethical approval or approval by research review committee, confirmed with reference number	14

Citation: Eldridge SM, Chan CL, Campbell MJ, Bond CM, Hopewell S, Thabane L, et al. CONSORT 2010 statement: extension to randomised pilot and feasibility trials. BMJ. 2016;355. This is an Open Access article distributed in accordance with the terms of the Creative Commons Attribution (CC BY 3.0) license (http://creativecommons.org/licenses/by/3.0/), which permits others to distribute, remix, adapt and build upon this work, for commercial use, provided the original work is properly cited.

*We strongly recommend reading this statement in conjunction with the CONSORT 2010, extension to randomised pilot and feasibility trials, Explanation and Elaboration for important clarifications on all the items. If relevant, we also recommend reading CONSORT extensions for cluster randomised trials, non-inferiority and equivalence trials, non-pharmacological treatments, herbal interventions, and pragmatic trials. Additional extensions are forthcoming: for those and for up-to-date references relevant to this checklist, see www.consort-statement.org.

CONSORT 2010 Flow Diagram

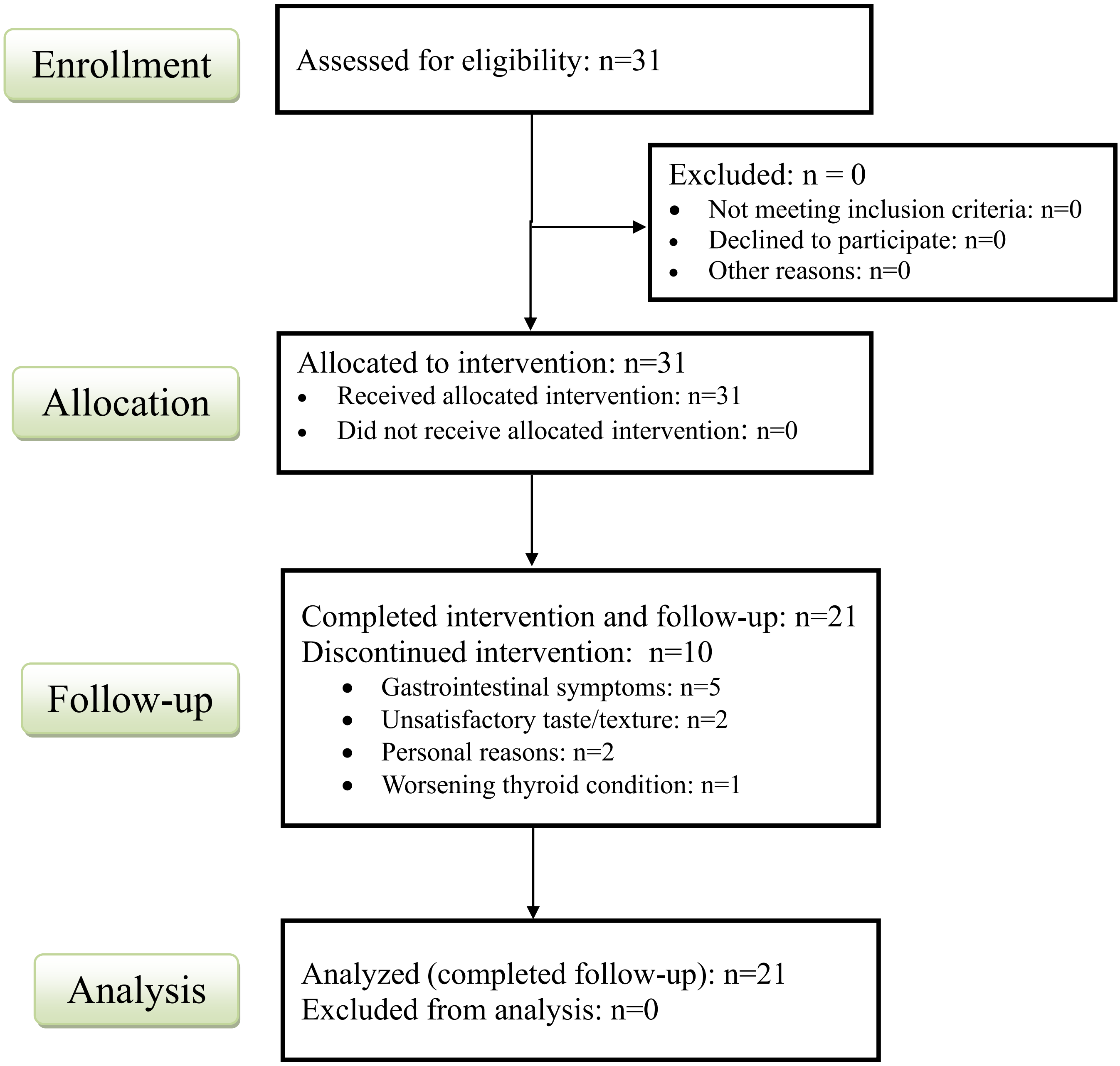

CONSORT 2010 flow diagram illustrating the progress of participants through each stage of the pilot clinical trial, including enrollment, allocation, follow-up, and analysis.
