# Supplementary material for: Initial allergenicity assessment of Ulva sp. seaweed flour
Source: NPJ Sci Food. 2025 Dec 3;10:1. doi: 10.1038/s41538-025-00638-x (PMC12764789; doi:10.1038/s41538-025-00638-x)
Supplement: Supplementary file 2 — Supplementary Data1 [file 41538_2025_638_MOESM2_ESM.pdf]

Supplementary Data 1. Ulva-DBF Food Allergen Homology Assessment Summary (Compared to AOL and Allergen.org)

Allergen counter	Allergen Source	Allergen Biochemical Name	Trial Test Group	Trial Identification Methods	Trial Control Group	Trial Results Supporting Identification of New Allergen	Is the Allergen a Pan-Allergen?	Is the Allergen Evolutionarily Conserved?	What Is the Biological Role of the Allergen?	Trial Clinical Significance for Ulva-DBF Allergenicity	Comments	REFERENCES
1	Striped catfish	Glyceraldehyde-3-phosphate dehydrogenase (GAPDH)	77 children, ages 1 to 18 years, had clinically confirmed fish allergies and a documented history of IgE-mediated symptoms	SPT, Immunoblotting, MS analyses	4 serum donors: 2 nonatopic and 2 atopic individuals tolerant to fish	6% had IgE binding to raw catfish GAPDH[1]		Highly conserved protein across various organisms[2]	Catalyzes the conversion of glyceraldehyde 3-phosphate to glycerate-1, 3-biphosphate, generating NADH[2]	Unknown		1. doi:https://doi.org/10.1111/all.14574<br>2. doi:10.3389/FMICB.2020.00818/FULL
2	Peanuts	Cyclophilin	124 United States participants, (57% males), had positive ImmunoCAP test results for peanuts	ImmunoCAP, IgE inhibition experiment, Chromatographic methods, Recombinant allergen	Negative test results (<0.35 kUA/L) to all of Ara h 1, 2, 3, 6, 8 and 9	In 5 of 15 sera, ImmunoCAP assays detected IgE binding to Ara h 18 after preincubation[3]	Yes[4]	Highly conserved protein[5,6]	Cyclophilins, found in all cells, including prokaryotes and eukaryotes, are immunophilins[5,6]	Unknown		3. doi:10.1111/CEA.13833<br>4. doi:10.1002/1521-4141<br>5. doi:10.1186/GB-2005-6-7-226/FIGURES/3<br>6. doi:10.1038/s41598-023-40720-6
3	Salmon	Beta-Enolase	62 individuals with a clinical history of fish allergy underwent SPT and specific IgE testing	Microsequencing, Immunoblot, ELISA, and mediator release assay (sensitizing humanized rat basophil leukemia (RBL))	10 non-atopic serum samples. Protein identified by immunoblot using a specific anti-enolase	24% of the patients exhibited IgE reactivity to salmon enolase[7]		Highly conserved protein[8]	Abundant cytosolic enzymes, serving as a vital glycolytic metalloenzyme, play a crucial role in energy metabolism[8]	Unknown		7. doi:10.1111/cea.12117<br>8. doi:10.1016/j.jaip.2021.04.005
3	Yellowfin tuna	Beta-Enolase	62 individuals with a clinical history of fish allergy underwent SPT and specific IgE testing	Microsequencing, Immunoblot, ELISA, and mediator release assay (sensitizing humanized RBL)	10 non-atopic serum samples. Protein identified by immunoblot using a specific anti-enolase	19% of the patients exhibited IgE reactivity to tuna enolase[7]		Highly conserved protein[8]	Abundant cytosolic enzymes, serving as a vital glycolytic metalloenzyme, play a crucial role in energy metabolism[8]	Unknown		
3	Striped catfish	Beta-Enolase	77 children (under 18 years), with clinically confirmed fish allergies and a documented history of IgE-mediated symptoms	SPT, Immunoblotting, MS analyses	4 Serum donors: 2 nonatopic and 2 atopic individuals tolerant to fish	21% had IgE binding to raw catfish Beta-Enolase[1]		Highly conserved protein[8]	Abundant cytosolic enzymes, serving as a vital glycolytic metalloenzyme, play a crucial role in energy metabolism[8]	Unknown		
3	Chicken	Beta-Enolase	7 individuals, exclusively allergic to chicken meat and sourced from Europen clinical centers, with documented allergies, positive skin prick tests (SPT), and specific IgE reactivity. Symptoms related to bird feathers and chicken eggs were excluded	Edman sequencing and MS analysis, ELISA, SPT	The detection of enolase presence by specific antibodies within chicken leg extract	6 of 7 patients had specific IgE to enolase chicken Gal d 9[9]		Highly conserved protein[8]	Abundant cytosolic enzymes, serving as a vital glycolytic metalloenzyme, play a crucial role in energy metabolism[8]	Unknown		9. doi:10.1111/ALL.12968
4	Pistachio	Manganese superoxide dismutase (MnSOD)	25 individuals exhibiting symptoms such as oral allergy syndrome, gastrointestinal discomfort, and skin itching after ingestion, with a positive SPT	Recombinant allergen, ELISA, and immunoblotting assays	Three subjects who showed negative SPT responses and no specific IgE to pistachio nut extract	10 (40%) out of the 25 showed IgE binding to purified Pis v 4 on immunoblot[10]	yes[10]	Highly conserved across a wide range of organisms[11]	Defense protein from iron/MnSOD family of proteins[12]	Unknown		10. doi:10.2332/ALLERGOLINT.10-OA-0174<br>11. doi:10.1080/10408398.2017.1379947<br>12. doi:10.1074/jbc.m310623200
5	Catfish	Glucose 6-phosphate isomerase	77 children, ages 1 to 18 years, with clinically confirmed fish allergies and documented history of IgE-mediated symptoms	SPT, Immunoblotting, MS analyses	4 Serum donors: 2 nonatopic and 2 atopic individuals tolerant to fish.	8% had IgE binding to raw catfish GAPDH[1]		Highly conserved protein across various organisms[13]	Involved in glycolysis, converting glucose-6-phosphate to fructose-6-phosphate, and acts as an angiogenic and neurotrophic factor[13]	Unknown		13. doi:10.20463/JENB.2019.0014
6	Salmon	Aldolase A	62 individuals with a clinical history of fish allergy underwent SPT and specific IgE testing	Microsequencing, Immunoblot, ELISA, and mediator release assay (sensitizing humanized RBL)	10 non-atopic serum samples. Protein identified by immunoblot using a specific anti-enolase	26% of patients exhibited IgE reactivity to raw salmon aldolase (0.4–13.0 kUA/L, median 4.5 kUA/L)[7]		Highly conserved across a wide range of organisms[14]	A ubiquitous cytosolic enzyme that catalyzes the fourth step of glycolysis[14]	Unknown		14. doi:10.3389/FMOLB.2021.719678/BIBTEX
6	Tuna	Aldolase A	62 individuals with a clinical history of fish allergy underwent SPT and specific IgE testing	Microsequencing, Immunoblot, ELISA, and mediator release assay (sensitizing humanized RBL)	10 non-atopic serum samples. Protein identified by immunoblot using a specific anti-enolase	13% of the patients exhibited IgE reactivity to tuna aldolase(0.5–8.0 kUA/L, median 2.8 kUA/L)[7]		Highly conserved across a wide range of organisms[14]	A ubiquitous cytosolic enzyme that catalyzes the fourth step of glycolysis[14]	Unknown		
6	Catfish	Aldolase A	77 children, ages 1 to 18 years,with clinically confirmed fish allergies and documented history of IgE-mediated symptoms	SPT, Immunoblotting, Mass Spectrometric analyses	4 Serum donors: two nonatopic and two atopic individuals tolerant to fish.	21% had IgE binding to raw catfish aldolase A[1]		Highly conserved across a wide range of organisms[14]	A ubiquitous cytosolic enzyme that catalyzes the fourth step of glycolysis[14]	Unknown		
7	Black tiger shrimp	Glycogen phosphorylase-like protein	85 individuals diagnosed with shrimp allergy, either through challenge tests or physician diagnosis, in Hong Kong and Thailand.	Western blotting, MS, ELISA with recombinant shrimp allergens	10 sera of Non-atopic individuals from Hong Kong were pooled	8/17 47.1% showed IgE binding to recombinant shrimp glycogen phosphorylase[15]		Commonly conserve active sites[16]	Glycogen phosphorylase facilitates glycogen breakdown through the removal of glucose units[17]	Unknown		15. doi:10.1111/ALL.15370<br>16. doi:10.1006/JMBI.1993.1621<br>17. doi:10.1128/JB.01566-05
8	Banana Prawn	Enolase	21 participants from Thailand (13 male, 8 female, ages 4 to 41 years), with allergic reactions to shrimp, underwent SPT for food allergens and/or had serum IgE levels quantitated	Immunoblotting, and Mass Spectrometry	Serum samples were collected from healthy individuals	IgE binding to enolase was detected in banana shrimp muscle[18]	Yes (pollen and plant-based foods)[89]	Consistently preserved across various taxonomic groups of organisms[19]	A common cytosolic enzyme and a notable glycolytic metalloenzyme[8]	Unknown		18. doi:10.1016/j.anai.2014.06.002<br>19. doi:10.1016/J.MOLIMM.2023.03.012

9	Chicken	Myosin light chain	33 participants from Austria and Spain, were diagnosed with poultry meat allergy. Two distinct IgE reactivity profiles were identifiable	Immunoblot, Peptide Mass Fingerprinting, Recombinant allergens, ELISA	Serum from individuals who were either non-allergic or allergic to allergens other than poultry meat allergens	77% of 28 patients sera diagnosed with genuine chicken meat allergy (group 2) tested positively for rGal d 7, indicating that Gal d 7 likely serves as a primary chicken meat allergen[20]		Highly conserved across different species[21]	Calmodulin family members play a pivotal role in the mechanical and enzymatic functions of the myosin holoenzyme[21]	Unknown	Plants do not produce myosin light chain	20. doi:10.1016/j.jaci.2020.02.033<br>21. doi:10.1080/19490992.2015.1054092
9	Red swamp crayfish	Myosin light chain	7 participants from China were diagnosed with crustacean allergies	MS, Western Blot and Dot Blot Analysis, Indirect and Competitive Inhibition ELISA, Recombinant allergens	Serum samples from nonallergic individuals	All participants had IgE binding to natural Pro c 5 by ELISA[22]		Highly conserved across different species[21]	Calmodulin family members play a pivotal role in the mechanical and enzymatic functions of the myosin holoenzyme[21]	Unknown	Plants do not produce myosin light chain	22. doi:10.1021/ACS.JAFC.5B01318
9	Shrimp	Myosin light chain	31 participants, mostly from Central Europe, with confirmed shrimp allergies	Immunoblotting, Recombinant production, MS, ImmunoCAP	Non-atopic subject	6/31 (19%) had IgE binding results to Cra c 5[23]		Highly conserved across different species[21]	Calmodulin family members play a pivotal role in the mechanical and enzymatic functions of the myosin holoenzyme[21]	Unknown	Plants do not produce myosin light chain	23. doi:10.1016/J.MOLIMM.2011.06.216
9	Green mud crab	Myosin light chain	10 Subjects allergic to crab	Recombinant production, iELISA, Dot Blot Analysis, BAT, Hman LAD2 mast cell degranulation assay	2 non-allergic individuals	All had IgE-binding activity by dot blot[24]		Highly conserved across different species[21]	Calmodulin family members play a pivotal role in the mechanical and enzymatic functions of the myosin holoenzyme[21]	Unknown	Plants do not produce myosin light chain	24. doi:10.1021/ACS.JAFC.9B04294
9	Black tiger shrimp	Myosin light chain	38 participants exhibited shrimp allergies and had elevated IgE levels specific to shrimp	Dimensional Electrophoresis, Immunoblotting, MS and Edman sequencing, Recombinant protein	A nonatopic subject	Myosin light chain was recognized by Western blot analysis in 21 out of 38 participants, representing 55% of the cohort[25]		Highly conserved across different species[21]	Calmodulin family members play a pivotal role in the mechanical and enzymatic functions of the myosin holoenzyme[21]	Unknown	Plants do not produce myosin light chain	25. doi:10.1016/J.JACI.2008.07.023
9	Black tiger shrimp	Myosin light chain	85 individuals diagnosed with shrimp allergy, either through challenge tests or physician diagnosis, in Hong Kong and Thailand	Western blotting, MS, ELISA Recombinant allergens	The sera of 10 subjects from Hong Kong were pooled to serve as controls, selected from non-atopic individuals.	2/17 11.8% had IgE binding to recombinant shrimp Myosin light chain[15]		Highly conserved across different species[21]	Calmodulin family members play a pivotal role in the mechanical and enzymatic functions of the myosin holoenzyme[21]	Unknown	Plants do not produce myosin light chain	
10	Barramundi	Collagen alpha	75 children (<18 years old) and 26 adults with established clinical fish allergies underwent ImmunoCAP and/or SPT	ELISA, immunoblots, BAT, MS	2 nonatopic individuals with no history of type I allergy and 3 atopic individuals with allergies other than to fish	Among the 20 fish-allergic individuals sensitized to collagen who lacked parvalbumin-specific IgE, 8 had IgE binding to collagen from specific fish species[26]	Yes[27]	Consistently preserved[28]	The predominant component of the extracellular matrix in mammals[28]	Unknown	Plants do not produce collagen alpha	26. doi:10.1016/J.JAIP.2020.04.063<br>27. doi:10.1111/ALL.12836<br>28. doi:10.1007/S13205-019-1616-9/FIGURES/4
10	Salmon	Collagen alpha	75 children (under 18 years old) and 26 adults with established clinical fish allergies underwent ImmunoCAP and/or SPT	ELISA, immunoblots, BAT, MS	2 nonatopic individuals without a history of type I allergy and 3 atopic individuals with allergies other than to fish	Among the 20 fish-allergic individuals sensitized to collagen who lacked parvalbumin-specific IgE, eight showed IgE binding to collagen from specific fish species[26]	Yes[27]	Consistently preserved[28]	The predominant component of the extracellular matrix in mammals[28]	Unknown	Plants do not produce collagen alpha	
11	Shrimp	Troponin C	31 participants, mostly from Central Europe, with confirmed shrimp allergies	Immunoblotting, Recombinant production, MS, ImmunoCAP	Non-atopic subject	9/31 (29%) had IgE-positive binding results to Cra c 6[23]		Relatively high conservation[29]	Crucial for determining the functional characteristics of striated muscle[29]	Unknown	Plants do not produce Troponin C	29. doi:10.1152/PHYSIOLGENOMICS.00197.2007
11	Parasitic fish worm	Troponin-like protein								Unknown	Plants do not produce troponin-like protein	
12	Clam, Mollusc, Kamchatka crab, cuttlefish, bigfin reef squid, red squid, Octopus, Disk abalone	Tropomyosin	35 participants with allergic symptoms to *Dermatophagoides pteronyssinus* (mites) underwent SPT along with DBPCFC, OFC, or a combination of both; 7 participants confirmed allergy to shrimp	ImmunoCAP and ELISA	28 participants had negative OFC for shrimp. 35.7% (10/28) had positive SPT. 7.1% (2/28) exhibited IgE reactivity to shrimp tropomyosin while 25% (7/28) showed IgE reactivity to shrimp	5/7 (71.4%), tested positive for IgE binding to shrimp tropomyosin[30]	Invertebrate pan allergen found in crustaceans and arachnids[31,32]	Conserved protein, particularly among vertebrates[33]	It serves as both a stabilizer for actin filaments and a controller of muscle contraction[33]	Unknown	Plants do not produce tropomyosin	30. doi:10.1016/J.JACI.2009.11.043<br>31. doi:10.2500/AAP.2021.42.210057<br>32. doi:10.1016/j.jaci.2022.12.827<br>33. doi:10.1002/CM.20152
12	Noble scallops, North Pacific Krill, Krill	Tropomyosin					Invertebrate pan allergen found in crustaceans and arachnids[31,32]	Conserved protein, particularly among vertebrates[33]	It serves as both a stabilizer for actin filaments and a controller of muscle contraction[33]	Unknown	Plants do not produce tropomyosin	

12	Parasitic roundworm	Tropomyosin	356 individuals who reported both current and past symptoms of asthma (asthmatics)	ImmunoCAP, ELISA, Recombinant Allergens	435 controls that had never experienced allergy symptoms.	A notable correlation observed with sensitization to the highly allergenic and cross-reactive tropomyosins, particularly Asc l 3[34]	Invertebrate pan allergen found in crustaceans and arachnids[31,32]	Conserved protein, particularly among vertebrates[33]	It serves as both a stabilizer for actin filaments and a controller of muscle contraction[33]	Unknown	Plants do not produce tropomyosin	34. doi:10.1111/CEA.12513
12	Japanese flying squid	Tropomyosin	4 participants exhibited immediate hypersensitivity reactions after consuming squid, and 7 American participants showed sensitivity to shrimp but had minimal or no prior exposure to squid as a dietary item, were identified	Column chromatography, immunoblotting, Mouse Polyclonal and Monoclonal Antibodies, Amino acid sequence analyses		All 11 patients tested had IgE binding to purified Tod p 1[35]	Invertebrate pan allergen found in crustaceans and arachnids[31,32]	Conserved protein, particularly among vertebrates[33]	It serves as both a stabilizer for actin filaments and a controller of muscle contraction[33]	Unknown	Plants do not produce tropomyosin	35. doi:10.1016/S0091-6749(96)80011-X
12	Parasitic fish worm	Tropomyosin	10 participants with proven allergy to Anisakis-contaminated seafood (group A), 62 participants with suspected allergy to Anisakis (group B), and 16 participants with household insect inhalant allergies	Immunoblotting	Titers of specific IgE in asymptomatic subjects	None of the 10 patients showed IgE binding to native or rAni s 3. Cross-reactivity with tropomyosins from other invertebrates is responsible for high titers of specific IgE in asymptomatic subjects[36]	Invertebrate pan allergen found in crustaceans and arachnids[31,32]	Conserved protein, particularly among vertebrates[33]	It serves as both a stabilizer for actin filaments and a controller of muscle contraction[33]	Unknown	Plants do not produce tropomyosin	36. doi:10.1034/J.1398-9995.2000.00734.X
13	Wheat	Thioredoxin	17 bakers with occupational asthma. 20 individuals diagnosed with grass allergy based on positive clinical history, SPT, and serum IgE to grass pollen, without clinical symptoms of wheat or maize allergy	Enriched wheat cDNA repertoire. ELISA	17 healthy participants	Among bakers with occupational asthma, the sensitization rate was 47%, while among participants with grass pollen allergy but no clinical history of cereal allergy, it was 35%[37]	Yes in yeast[38]	Antioxidant enzymes preserved throughout evolution[39]	Safeguard organisms against oxidative stress and participate in redox signaling[39]	Unknown		37. doi:10.1016/J.JACI.2005.11.040<br>38. doi:10.4049/JIMMUNOL.178.1.389<br>39. doi:10.3390/ANTIOX12040944/S1
14	Parasitic fish worm	Protein with unknown function	37 Anisakis simplex (fish parasite) allergic participants. 15 participants with allergies to fish, shrimp, and fruit	Recombinant Allergens, Immunoblotting	10 healthy participants	IgE-positive reactions to rAni s 11 were observed in 78% of cases[40]			Protein with unknown function	Unknown		40. doi:10.1159/000444981
15	Pea	Convicilin	18 pea allergic participants validated by a convincing clinical history, positive OFC, elevated specific serum IgE levels, and positive SPT	Immunoblot, Immunoblot Inhibition assays, Recombinant DNA	5 participants exhibited allergies to fish but not to pollens or plant foods	13/18 (72%) reacted with Pis s 2 from the crude extract on immunoblot[41]		Conserved domains in mammalian[42]	Seed storage proteins[43]	Unknown	Potential major allergens from pea	41. doi:10.1111/J.1365-2222.2004.02085.X<br>42. doi:10.1515/BC.2007.028<br>43. doi:10.1042/BJ1910509
16	Parasitic fish worm	SXP/RAL-2 family protein	36 participants with Anisakis allergy confirmed by compelling clinical history and elevated levels of specific serum IgE	Recombinant DNA, Immunoblotting assays. Immunoblot inhibition assays.	5 control participants with negative results in CAP testing for Anisakis	5/36 participants exhibited reactivity to both the natural and recombinant forms of Ani s 9 as determined by immunoblotting[44]		Evolutionarily conserved[45,46]	Vesicular transport	Unknown	https://allergen.org/viewallergen.php?aid=56	44. doi:10.1016/J.MOLBIOPARA.2008.02.008<br>45. doi:10.1186/1471-2148-8-142/TABLES/4<br>46. doi:10.18632/ONCOTARGET.10957
17	peach	Thaumatin-like protein (TLPs)	14 male, 17 female participants from Spain, ages 16 to 46 years, were identified with peach allergy, validated by compelling clinical history, positive SPT, and positive OFC, excluding those with previous anaphylaxis	Recombinant protein production, inhibition immunoblots, enzyme-linked immunosorbent inhibition assays, BAT	36 participants with dust mite allergies, not displaying allergies to plant foods or pollen	Pru p 2 was detected in 77% of the analyzed sera using ELISA, while over 80% of patients showed reactivity when BAT or SPTs were conducted[47]	Sten -allergens [48]		TLPs are linked with host defense and developmental processes across plants, animals, and fungi[49]	Unknown	https://allergen.org/viewallergen.php?aid=664	47. doi:10.1111/J.1365-2222.2010.03578.X<br>48. doi:10.1186/1710-1492-6-1<br>49. doi:10.2174/1389203720666190318164905
18	Parasitic fish worm	Paramyosin	26 participants with allergic symptoms to marinated fish had positive results in SPT using an *A. simplex* extract, along with positive CAP results	Constructed a cDNA library. Screened using rabbit anti-*A. simplex* hyperimmune serum, followed by human sera. ELISA	5 atopic participants with negative results for *A. simplex* but elevated specific IgE levels to prevalent aeroallergens	6/26 participants tested positive for the recombinant protein, while 23/26 (88%) tested positive for the native protein[50]	Yes[51]	Conserved C-terminal proteins[52]	A significant myofibrillar protein found in smooth muscle among mollusks[52]	Unknown	Less explored in plants compared to invertebrates	50. doi:10.1159/000024442<br>51. doi:10.1016/J.MOLIMM.2018.04.008<br>52. doi:10.3390/BIOLOGY11030453
18	Rapana venosa	Paramyosin	5 participants with sea snail allergies exhibited specific IgE to *R. venosa* paramyosin	Immunoblotting, Recombinant production, Western blot, and dot blot tests	3 participants with shrimp allergies and healthy individuals were included	All participants with sea snail allergies had a reaction to the target protein[53]		Conserved C-terminal proteins[52]	A significant myofibrillar protein found in smooth muscle among mollusks[52]	Unknown		53. doi:10.1021/ACS.JAFC.0C04418
18	Disk abalone	Paramyosin	18 participants tested positive for crustacean allergies via CAP-RAST tests, specifically for shrimp, crab, or mollusk	Recombinant production, ELISA and inhibition ELISA, Immunoblotting, inhibition immunoblotting, Protein Assay Kit	19 healthy participants	16 /18 participants exhibited positive reactivity to disc abalone paramyosin[54]		Conserved C-terminal proteins[52]	A significant myofibrillar protein found in smooth muscle among mollusks[52]	Unknown		54. doi:10.1016/J.FOODCHEM.2010.07.020
19	Peanut	Pathogenesis-related protein, PR-10, Bet v 1 family member	20 participants from Switzerland and the Netherlands, diagnosed with peanut allergy, were confirmed positive through a DBPCFC to peanut	Recombinant production, Basophil histamine release assay. RAST inhibition, EAST inhibition, Immunoblotting and Immunoblot inhibition	A subject devoid of pollen and food allergies	All participants had oral cavity symptoms during DBPCFC, with 40% experiencing more severe reactions. Specific IgE to rAra h 8 was detected by CAP-FEIA, and in 5 of 7 participants tested, basophil histamine release exceeded 20%[55]	Yes[51]	Structural and functional conservation across plant species[56]	Pathogenesis-related protein[56]	Unknown		55. doi:10.1016/j.jaci.2004.09.014<br>56. doi:10.1371/JOURNAL.PONE.0095102
20	Flat fish, whiff	Beta-parvalbumin	10 children ages 1 to 3 years and 6 adults ages 21 to 36, tested positive for fish allergies via ImmunoCAP and SPT tests for various fish species, with negative results for *Anisakis simplex*	Recombinant production, Immunoblotting, ELISA inhibition		15/16 participants showed positive reactivity to whiff parvalbumin[57]	Yes[58]	Structural and functional conservation, especially within vertebrates[59]	A calcium-binding protein found in muscles[59]	Unknown	Plants do not produce parvalbumin; https://allergen.org/viewallergen.php?aid=408	57. doi:10.1111/J.1398-9995.2009.02162.X<br>58. doi:10.1016/j.jaci.2010.12.137<br>59. doi:10.3389/FNCIR.2023.1297643/BIBTEX

21	Turnip	Prohevein homologue	29 women, 5 men, ages 18 to 64 years, and 11 girls, 15 boys, ages 0 to 16 years, previously identified as allergic to natural rubber latex (NRL), were tested for IgE antibodies against prohevein (For IgE-ELISA)	Immunoblotting and immunoblot inhibition, ELISA, ELISA inhibition and SPT	62 participants, consisting of 40 women and 22 men ages 2 to 64 years, without NRL allergy	51/60 (82%) participants had IgE binding to purified Bra r 2 in ELISA and 4/6 participants had a positive SPT to purified Bra r 2[60]			Associated with stress and pathogenic responses[61]	Unknown	Plants do not produce Prohevein	60. doi:10.1016/S0091-6749(99)70135-1<br>61. doi:10.1016/J.BIOCHI.2016.06.006
22	European hazelnut	Profilin	65 participants from Switzerland and Germany with allergic history to hazelnuts (aged 18 to 68 years, 13 male). 39 participants underwent SPT with hazelnut extract and part underwent hazelnut prick-to-prick tests. All were positive for DBPCFC	Recombinant production, ImmunoCAP, Immunoblotting, Basophil histamine release	For basophil histamine release, a non-allergic serum was used as a negative control	10/65 participants immunoblot showed IgE binding to rCor a 2[62]	Yes[48]	Highly conserved[63]	Regulating actin polymerization dynamics, particularly during cell motility[63]	Unknown		62. doi:10.1042/BJ20041062<br>63. doi:10.1016/j.tcb.2004.07.003

Abbreviations: AOL, AllergenOnline; BAT, Basophil Activation Test; DBPCFC, Double-Blind, Placebo-Controlled Food Challenges; GAPDH, Glyceraldehyde-3-phosphate dehydrogenase; UlvaLe, land-based cultivation system to produce Ulva sp.
References are listed in column S and in the supplementary file titled "Supplementary Data 1. References."
